# Dataset of human medial temporal lobe single neuron activity during declarative memory encoding and recognition

**DOI:** 10.1038/sdata.2018.10

**Published:** 2018-02-13

**Authors:** Mailys C. M. Faraut, April A. Carlson, Shannon Sullivan, Oana Tudusciuc, Ian Ross, Chrystal M. Reed, Jeffrey M. Chung, Adam N Mamelak, Ueli Rutishauser

**Affiliations:** 1 Department of Neurosurgery, Cedars-Sinai Medical Center, Los Angeles, CA 90048, USA; 2 Division of Biology and Biological Engineering, California Institute of Technology, Pasadena, CA 91125, USA; 3 Department of Neurosurgery, Huntington Memorial Hospital, Pasadena, CA 91125, USA; 4 Department of Neurology, Cedars-Sinai Medical Center, Los Angeles, CA 90048, USA; 5 Computation and Neural Systems Program, California Institute of Technology, Pasadena, CA 91125, USA; 6 Center for Neural Science and Medicine, Department of Biomedical Science, Cedars-Sinai Medical Center, Los Angeles, CA 90048, USA

**Keywords:** Neurology, Long-term memory, Hippocampus

## Abstract

We present a dataset of 1,576 single neurons recorded from the human amygdala and hippocampus in 65 sessions from 42 patients undergoing intracranial monitoring for localization of epileptic seizures. Subjects performed a recognition memory task with pictures as stimuli. Subjects were asked to identify whether they had seen a particular image the first time (‘new’) or second time (‘old’) on a 1–6 confidence scale. This comprehensive dataset includes the spike times of all neurons and their extracellular waveforms, behavior, electrode locations determined from post-operative MRI scans, demographics, and the stimuli shown. As technical validation, we provide spike sorting quality metrics and assessment of tuning of cells to verify the presence of visually-and memory selective cells. We also provide analysis code that reproduces key scientific findings published previously on a smaller version of this dataset. Together, this large dataset will facilitate the investigation of the neural mechanism of declarative memory by providing a substantial number of hard to obtain human single-neuron recordings during a well characterized behavioral task.

## Background & Summary

Rapidly forming new memories and to later retrieve and describe these memories is an essential component of cognition. In mammals, the medial temporal lobe (MTL) memory system^1,2^ enables the formation and retrieval of one kind of such rapidly forming memory: declarative or episodic, which are memories of events or facts that can be consciously recalled and described^3,4^. The MTL encompasses both cortical- and subcortical brain areas and includes the hippocampus, amygdala and the perirhinal, entorhinal, and parahippocampal cortices^1^. Much of the fundamental work to decipher the underlying mechanisms of declarative memory has relied on extracellular recordings of single neurons and local field potentials in animals implanted with electrodes^5^. While this has revealed an extensive body of knowledge, the corresponding mechanisms in humans remain, in comparison, relatively poorly understood for a lack of similar invasive brain access. It is important to develop a similar depth of understanding directly in humans because aspects of declarative memories are thought to be unique to humans and/or are challenging to study in animals^6^ or non-invasively in humans. While rarely possible, in some clinical situations it is possible to perform such recordings in humans^7^. These extracellular recordings performed in human epilepsy patients implanted with depth electrodes have provided crucial information at the single-cell level and have revealed critical aspect of memory function, including the existence of category^8^, concept^9,10^, and face cells^11,12^, cells encoding spatial context^13^, novelty-and familiarity modulated cells^14–17^, persistent activity during working memory and free recall^18–20^, and the formation of episodic memories^21^.

**Figure 1:**
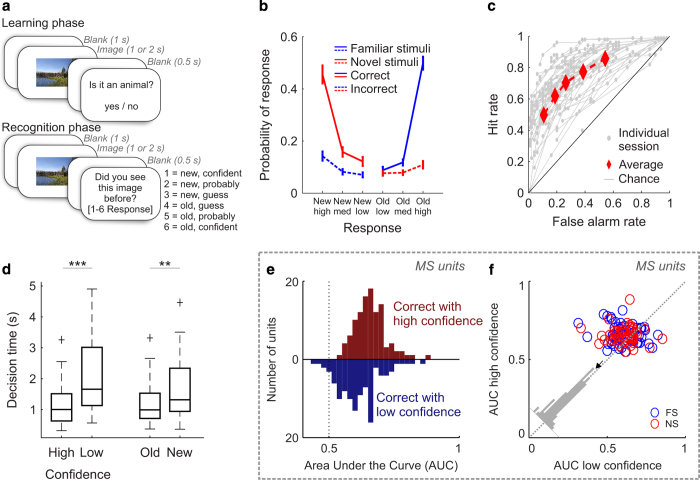
The recognition memory task, behavioral results and MS single-neuron ROC analysis. (**a**) the task is composed of a learning phase during which 100 new images are shown to subjects who have to report whether it showed an animal or not; and a recognition test phase showing both new and old images to subjects who indicate whether they saw it or not before by reporting their confidence level. Stimuli were displayed on the screen for either 1 or 2 s (see usage notes). (**b**–**d**) Behavior. (**b**) Probability of responses, conditional on the ground truth (red or blue). At all levels of confidence, subjects were more likely to be correct than incorrect (straight and dashed lines, respectively). (**c**) Behavioral ROC curve for individual sessions (gray) and average (red). Each data point is a different confidence response. (**d**) Decision times, relative to Question screen onset. Correct high confidence decisions were made faster than low confidence decisions, and old decisions faster than new decisions. Boxplots represent quartiles (25%, 75%), line is median, whiskers show range up to 1.5 time the interquartile range, and dots above whiskers show outliers. Trials with decision times larger than 5 s were excluded from this analysis. (**e**,**f**) Single-neuron ROC analysis. The response of MS neurons was modulated by confidence. (**e**) AUC for MS neurons for high (red) and low (blue) confidence (*n*=107 units, the 2 distributions were significantly different, Bootstrap with 1,000 runs, *P*<10−3). (**f**) Pairwise comparison of AUC values. For 73 of 107 units, the AUC was high>low (*P*<0.001, sign test). The average difference was above the diagonal (inset). FS: familiarity selective neurons, NS: novelty selective neurons. A MS neuron was FS if the mean of all familiar trials was larger than all novel trials and NS otherwise.

One of the types of experiments used frequently to study declarative memory is the new/old recognition memory experiment, a type of experiment that has been used extensively in human fMRI^22^, scalp EEG^23^, and single-neuron recordings^24^ as well as in non-human primates^25^. Here, we describe a large publicly released human single-neuron dataset that consist of 633 and 943 neurons recorded from the hippocampus and amygdala, respectively. These neurons were recorded in 42 patients that performed the new/old recognition memory experiment (Fig. 1). Stimuli were images of animals, objects or other complex natural scenes, each of which subjects saw either only once or twice. The first time, the image was ‘new’, whereas the second time it was ‘old’. For each image, subjects were asked whether they had seen the image before or not and with what confidence (1–6 confidence scale). Along with the neuronal recordings, we provide detailed behavioral data and technical validation of the quality of the isolated neurons. Together, this large dataset of rare direct recordings from single neurons of behaving humans will allow investigators to explore the mechanisms of recognition memory at the single-neuron level. This dataset, which is an extended version with new data added (22 sessions are unpublished) of what was used in^14,15^, has extensive reuse value by, for example, allowing direct tests of predictions made by memory models^26,27^ or by facilitating population-level analyses of neuronal dynamics^28–30^ that require hundreds of neurons to become feasible.

## Methods

The methods used to acquire and process this dataset have been published previously^14,18^. For convenience, we here provide a brief but comprehensive description of all steps.

### Subjects

Subjects were 42 individuals (see Table 1) who were patients with intractable localization-related epilepsy who underwent depth electrode monitoring for localization of their seizure focus as preparation for potential surgical resection. All patients volunteered to participate in this study and provided written informed consent. All released data is de-identified and linked only by a subject identifier. Note that subject identifiers are not continuous because not all subjects performed this experiment. Electrode locations were chosen according to clinical criteria alone. All protocols were approved by the Institutional Review Boards of the California Institute of Technology, the Huntington Memorial Hospital, and Cedars-Sinai Medical Center.

### Electrodes and data acquisition

All recordings were performed with macro-micro depth electrodes available commercially (AdTech Medical Inc). Each such macro-electrode contained eight 40 μm diameter microwires^31^. The signal from each microwire was locally referenced to one of the eight microwires, thus allowing the recording of activity from seven microwires in each area. Data was recorded broadband (0.1–9,000 Hz filter) sampled at 32 kHz using either an Atlas or Cheetah system (Neuralynx Inc). Channels with inter-ictal epileptic activity were excluded. Only electrodes localized to the hippocampus or amygdala were included.

### Spike detection and sorting

**Figure 2:**
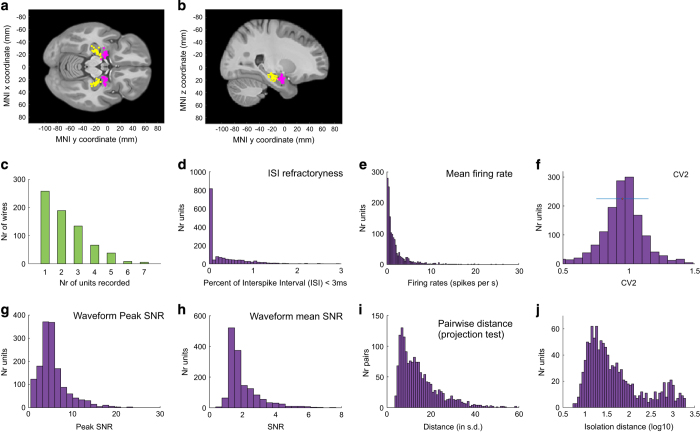
Electrodes placement and spike sorting and recording quality assessment. (**a**) Axial (z=−15) and (**b**) sagittal (x=25) views of template structural MRI showing electrodes placement for areas of patients in which at least one usable unit was recorded (hippocampus: yellow, amygdala: pink). (**c**) Histogram of how many units were identified on each active wire (only wires with at least one unit identified are counted). (**d**–**j**) Metrics quantifying individual clusters. (**d**) Histogram of proportion of inter-spike intervals (ISIs) which are shorter than 3 ms. The large majority of clusters had less than 0.5% of such short ISIs. (**e**) Histogram of mean firing rates. (**f**) Histogram of CV2 values of all units. (**g**) Histogram of the SNR of the mean waveform peak of each unit. (**h**) Histogram of the SNR of the entire waveform of all units. (**i**) Pairwise distance between all possible pairs of units on all wires where more than 1 cluster was isolated. Distances are expressed in units of s.d. after normalizing the data such that the distribution of waveforms around their mean is equal to 1. (**j**) Isolation distance of all units for which this metric was defined (*n*=1,119, median 31.3).

For each channel, the continuously recorded raw signal was bandpass filtered 300–3,000 Hz. Spikes were detected by threshold crossings of an energy signal computed by convolving the filtered raw trace with a kernel of approximate width of an action potential^32,33^. All detected spikes were subsequently spike sorted using the semiautomatic template-matching algorithm OSort, that is available as open source^32^. We relied on the following criteria to identify clusters that represented putative single neurons: i) stability of firing rate over time, ii) violation of the refractory period, iii) shape of the ISI distribution, iv) shape of the waveform and v) separation from other clusters. Similar looking clusters were merged. All clusters that passed these criteria were subsequently stored and are provided as lists of timestamps. Also, for each provided cluster we computed a series of spike sorting quality metric to validate its properties (see technical validation for a list of metrics provided and Fig. 2).

### MRI processing and localization

Electrode localization was performed based on post-operative MRI scans that were performed after implantation of the electrodes. These scans were registered to pre-operative MRI scans using Freesurfer’s mri_robust_register^34^ to allow accurate and subject-specific localization. To summarize electrode positions and to provide across-study comparability we in addition also aligned the pre-operative scan to the MNI152-aligned CIT168 template brain^35^ using a concatenation of an affine transformation followed by a symmetric image normalization (SyN) diffeomorphic transform^36^. This procedure provided the MNI coordinates that are reported here for every recording location.

### Psychophysics

The task is identical to that utilized in ref. ^14^. There are three versions of the task (see Table 2), which are all identical except for the images shown. Each stimulus set contains images chosen from five different visual categories, with an equal number of instances chosen from each. The experiment consisted of two parts: a learning and a recognition block (see Fig. 1 for details). During the learning block, subjects were shown 100 novel images. Each image shown was unique and was only shown once (shown for either 1 or 2 s, see usage note). Subjects were instructed to carefully watch the images for a later memory test (explicit memory formation). As a control, subjects indicated after every learning trial whether the image showed an animal or not. During the recognition block, a random subset of 50 of these images was shown again (now ‘old’), randomly intermixed with a new set of 50 novel images. After each image, subjects were asked whether they had seen this identical image before (‘old’) or not (‘new’) and with what confidence. Subjects provided their answer on a 1–6 confidence scale as following: 1=new, very sure; 2=new, sure; 3=new, guess; 4=old, guess; 5=old, sure; 6=old, very sure. Patients provided their answers by pressing buttons on an external response box (RB-740, Cedrus Inc.). Subjects could only provide answers after the onset of the question screen. The question screen was displayed till the answer was given (no timeout) and there was no time pressure to respond quickly. Images shown were approximately 9°×9° degrees in size. The task was implemented in MATLAB using the Psychophysics toolbox^37^. The task ran on a notebook computer placed in front of the patient. Event Transistor-Transistor Logic (TTLs) pulses were sent to the acquisition system via parallel port.

### Identification of selective cells

**Figure 3:**
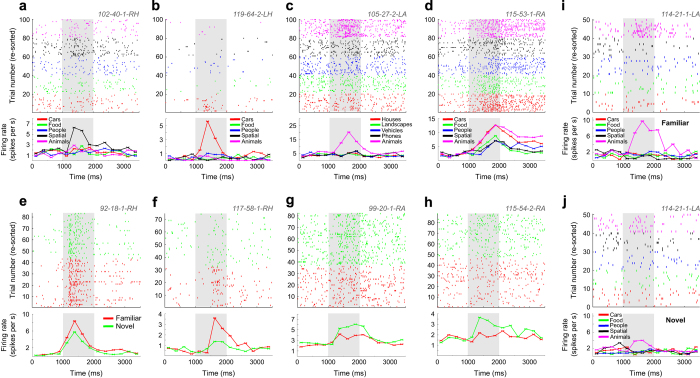
Example VS, MS and both VS and MS neurons. For each plot, the raster (top) and PSTH (bottom) is shown. Trials are re-sorted for illustration purposes. Visual identity (category) is indicated by color, and the legends show the corresponding label (variable). (**a**–**d**) VS neurons. Some units responded with a firing increase only to one category (**a**–**c**,**i**), whereas others showed a mixed response (**d**). Stimulus onset was at t=1,000 ms (gray). Significance of selection criteria (1×5 ANOVA) was 2×10−5 (**a**),5×10−7 (**b**), 8×10−11 (**c**), 3×10−11 (**d**). (**e**–**h**) MS neurons. MS units either responded with a firing increase for familiar stimuli (Familiarity selective neurons, **e**,**f**), or with an increase for novel stimuli (Novelty selective neurons, **g**,**h**). Significance of selection criteria (bootstrap test, familiar vs novel) was 0.04 (**a**), 0.001 (**b**), 0.02 (**c**), 0.02 (**d**). (**i**,**j**) Unit that qualifies as both a VS and MS neuron. Shown is the same unit, separately for when stimuli were familiar (**i**) and novel (**j**). ANOVA Category x NewOld: p category=2×10−12, p old/new=0.01, p interaction=3×10−5. Each unit is named with session ID, channel number, cell number and region abbreviation (e.g., 102-40-1-RH). PSTH bin size was 250 ms. Note that the visual category levels vary between cells because they belong to different variants of the experiment (see Table 2).

To demonstrate the use of the dataset and to replicate previous results in this larger dataset as a technical validation we here select for two types of selective cells using previously established criteria. First, we select for visually selective (VS) cells whose response after stimulus onset differentiates between the five visual categories of the images shown (see single-unit examples in Fig. 3a–d). Such cells were selected using a 1×5 ANOVA with *P*<0.05. The dependent variable was the firing rate in each of the 100 retrieval block trials counted in a 1.5 s large window that started 200 ms after stimulus onset. Second, we select for memory selective (MS) cells whose response differentiates between novel and familiar images (see single-unit examples in Fig. 3e–h). A cell qualified as an MS cell if its response in a 1.5 s window starting 200 ms after stimulus onset differed significantly between new and old stimuli that were correctly recognized (error trials were excluded; two-tailed, bootstrap comparison of means with 1,000 runs, *P*<0.05^14^).

### Code availability

All code is implemented using Matlab (Mathworks Inc.). Example code is provided as part of the dataset (Data Citation 1). We used Matlab versions R2015a, R2015b, and R2017a on Windows, Linux, and MacOS X, respectively.

## Data Records

The released data (Data Citation 1) consists of multiple parts, each of which is described in a separate section. All data files are stored as Matlab (*.mat) files. The data is organized as following: files which are common to all subjects (order in which stimuli were shown and stimuli themselves) are in the directory Stimuli. All data that varies session-by-session (behavior, recordings) is stored in session-specific directories. There are two such directories: events (which contains behavior) and sorted (which contains the spike times of identified neurons as well as some spike metrics).

### Events files

Events are timestamps that identify when patients pressed a button and when stimuli were shown (located in the ‘Data/events/’ directory). For each recording session, there is one ‘eventsRaw.mat’ file that lists all events for this session. This file has three columns: timestamp (in μS), TTL value, and experimentID. experimentID assigns a particular record to a given experiment as defined in defineNOSessions_release.m. TTL values are as following: Stimulus On (1), Stimulus Off (2), Question screen onset (3), Responses (31–36 for confidence 1–6, 20 and 21 for Yes and No during learning), end of delay after response (6), start of experiment block (55) and end of experiment block (66).

### Timestamps of sorted cells, waveforms and other extracellular recording metrics

For each channel x for which at least one putative single neuron was identified, a file Ax_cells.mat exists (located in the ‘Data/sorted/’ directory). The variable ‘spikes’ within this file contains one row for each detected spike on this channel. The columns of this variable are as following: clusterID, original ClusterID, Timestamp in μS, unused, original SpikeID. The unique number of clusterID entries determines how many neurons were identified on this channel. The original ClusterID and original SpikeID can be used to identify this spike in the raw file produced by the spike sorting algorithm OSort (not supplied in this data release). The variables ‘meanWaveform_learn’ and ‘meanWaveform_recog’ contain the mean waveform of all spikes of given cluster for the trials of the Learning and Test phases, respectively. Waveforms are provided in units of μV and are provided at a sampling rate of 100 kHz (256 datapoints are provided, equaling 2.56 ms in length). This up-sampling was performed to increase accuracy of the time of the spike peak, which improves alignment^33^. The variable ‘IsolDist_SNR’ contains for each cluster the value of the isolation distance (‘IsolDist’ field). The isolation distance quantifies, for every cluster, how far apart it is from the other clusters and the noise. We calculated the isolation distance in a ten-dimensional feature space (energy, peak amplitude, total area under the waveform and first five principal components of the energy normalizes waveforms)^38^. The calculation method for the isolation distance of a specific cluster requires that the number of spikes in this cluster does not exceed half of the total number of spikes^38^. For this reason, the isolation distance could not be calculated for some clusters. The variable ‘IsolDist_SNR’ also contains the signal-to-noise ratio (SNR) of the mean waveform of each cluster (‘SNR’ field).

### Stimulus order files

(see Table 2) There are two files that describe the stimuli shown to the subjects for each variant x (1–3) of the experiment (located in the ‘Code/dataRelease/stimFiles’ directory). The first file (newOldDelayStimuliX.mat) associates each stimulusID with a filename and visual category. There are three variables: fileMapping, categories, and categoryMapping. fileMapping(stimulusID) is a cell array that assigns a filename to each stimulusID. Categories(categoryID) is a cell array that assigns a name to each categoryID. categoryMapping is a matrix in which each row assigns a stimulusID (column 1) to a categoryID (column 2). The second file (NewOldDelayX_v3.mat) determines which images were shown and in which order. This is defined as lists of stimulusIDs. The information for the learning block is contained in the variable experimentStimuli(1).stimuliLearn, which is a list of stimuli IDS in order of presentation during the learning block. The information for the recognition block is contained in the variables experimentStimuli(2).stimuliRecog and experimentStimuli(2).newOldRecog. The variable ‘stimuliRecog’ lists the stimuliIDs in order of presentation as shown during the recognition block, whereas ‘newOldRecog’ specifies the ground truth of whether the image is new (0) or old (1).

### Images used as stimuli

(see Table 2) The images used for each variant of the experiment are contained in a separate directory as jpg files (located in the ‘Stimuli’ directory). The mapping between the filenames of the images and the stimuliID is specified in the stimulus file (see above).

### Brain area mapping

The brainArea.mat file specifies the mapping of channel numbers to brain areas for a given session (located in the ‘Data/events/’ directory). The file contains a matrix ‘brainArea’ in which each column maps a neuron to a brain area. The columns of brain area are: channelNr, clusterID, original clusterID, brainAreaID. The mapping of brainAreaID to brain areas is as following: 1=right hippocampus (RH), 2=left hippocampus (LH), 3=right amygdala (RA), 4=left amygdala (LA).

### Electrode position file

The electrodePos.xls file provides a list of electrode positions in MNI coordinates for each electrode on which at least 1 usable unit was recorded. MNI positions were determined based on post-operative MRI scans as summarized above (Fig. 2a,b). Note that due to subject-to-subject variability, MNI coordinates sometimes appear to place an electrode outside the boundaries of the hippocampus or amygdala on the template MNI brain. Note, however, that this is not necessarily the case since every brain is different and merging to an Atlas has inherent limitations. In cases where inspection of the raw (in subject space) MRI provided doubts as to the certainty of localization, we indicated this accordingly. The purpose of this is so that follow-up analysis can apply stricter criteria by potentially excluding these areas. We also provide a code that enables the user to exclude or include from analysis neurons according to whether they are recorded from within the epileptic focus or not (see *Description of code provided*).

## Technical Validation

### Behavior

Subjects performed a recognition memory test during which they had to rate 100 images as previously seen or not during a learning block (Fig. 1a). During the learning, subjects correctly categorized 96.8±0.9% of images (Animal yes/no). During the recognition block, subjects showed good memory: they correctly identified 70.4±14% of familiar images and reported only 26.5±16% of novel stimuli as false positive. Confidence ratings were systematically related to accuracy (Goodman-Kruskal gamma correlation, g=0.35±0.05, *t*-test versus chance *P*<10−7): the higher the confidence, the better the accuracy (Fig. 1b). This shows that the subjects were able to accurately judge their memory performance through confidence ratings, which is a hallmark of declarative memory^4^. In addition, we performed a Receiver operating characteristic (ROC) analysis to quantify the relationship between accuracy and confidence for each session (Fig. 1c). The average area under the curve (AUC) of the ROC was 0.77±0.09. The ROC was asymmetric (z-ROC slope=0.79, significantly less than 1, *P*<10−9), as expected for declarative memories^39^.

The decision time (time from question onset till response) varied systematically as a function of confidence and accuracy (repeated-measure ANOVA model). Correct high-confidence decisions were faster than low-confidence decisions (1.1±0.6 s versus 2.0±1.1 s, main effect of confidence F1,177=48.6, *P*<10−3; Fig. 1d, left). Correct familiar decisions were faster than correct novel decisions (1.2±0.6 s versus 1.6±0.8 s, main effect of familiarity, F1,177=10.1, *P*<10−3; Fig. 1d, right). Together, these behavioral results show that the subjects included in this dataset utilized declarative memory and were able to provide accurate subjective confidence ratings.

### Spike-sorting quality metrics

We next provide a detailed assessment of the quality of the recordings that are part of this dataset. For this purpose, we computed several spike sorting quality metrics for all units (Fig. 2) and for hippocampus and amygdala separately (Table 3): i) the number of units recorded per wire (Fig. 2c). The average yield per wire with at least one unit was 2.3 (range 1–7), ii) the percentage of inter-spike intervals (ISIs) below 3 ms was 0.29±0.47% (Fig. 2d), iii) the mean firing rate over all cells was 1.99±2.8 Hz (Fig. 2e), iv) the mean modified coefficient of variation of variability in the ISI (CV2) as in ref. 40 was 0.95±0.2 (P=0.81, not significantly different from 1, as expected from a Poisson process) (Fig. 2f), v) the ratio between the peak amplitude of the mean waveform of each cluster and the s.d. of the noise (peak SNR) was 5.77±3.85 (Fig. 2g), vi) the mean SNR was 2.06±1.14 (Fig. 2h), vii) the pairwise distance between all possible pairs of units on all wires with more than 1 cluster was isolated and the mean was 15.42±10.83 (Fig. 2i), and viii) the median isolation distance was 31.3 (Fig. 2j). We also computed the burst index (BI) which is equal to the proportion of ISIs less than 10 ms long and the mean across cells was 0.03±0.06 (Table 3). The spike width d (or trough-to-peak time) is the time between the trough and the point of time of maximal amplitude after the trough of the mean waveform (average of all spikes assigned to the cluster) (Table 3). The mean spike width was 0.59±0.01 and the distribution of spike widths was bimodal (*P*<4×10−4, Hartigan DIP test). This indicates that at least two different cell types were present in the population^14,41^. Together, this shows that the single neurons released as part of this dataset are well isolated and of high quality. Also, the provided quality metrics allow a further sub-selection for particularly high-quality units should this be a requirement of a particular analysis.

### Proportion of selective cells and their properties

To provide an assessment of tuning of neurons to the task, we next repeated our previously published selection for visually-and memory selective units^14^. Note that the previously published version was only on a subset of the data released here (22 sessions are new). Thus, this analysis constitutes a replication of our original result based on a larger dataset. Of the overall 1,576 recorded MTL neurons, 1,346 were considered. The remainder was excluded because of the following behavioral criteria (see ref. ^14^ for details). We excluded sessions with i) behavioral AUC <0.6 on all trials, ii) missing types of confidence responses, iii) below chance confidence judgments, and iv) less than 10 correct trials per condition. Of the 1,346 considered neurons, 250 (18.6%) neurons were classified as VS neurons (single-unit examples in Fig. 3a–d), 118 (8.8%) as MS neurons (Fig. 3e–h), and 20 (1.5%) as both VS and MS neurons (Fig. 3i,j) (see Table 4 for proportions per region). Single-neuron ROC analysis for every MS neuron and calculation of its AUC gives an estimation of its ability to encode the choice (novel or familiar) of a subject by counting spikes in an individual trial. The average AUC for all MS neurons, considering all correct trials, was 0.63±0.07. AUC values were also calculated using only high- or low-confidence trials. AUC values were significantly larger for high- than for low-confidence trials for all MS neurons together (0.66±0.06 versus 0.61±0.09, bootstrap comparison with 1,000 runs, *P*<10−3, Fig. 1e,f). This shows that the strength by which MS neurons signaled the familiarity of a stimulus was correlated with the confidence reported by the subject. Together, this analysis shows that this dataset contains VS and MS neurons with properties similar to those reported before. In particular, note that despite a substantially increased dataset size, the number of neurons that qualify as both a VS and MS neuron remains low and is compatible with the conclusion that the probabilities that a given cell qualifies as an MS or VS cell are independent of each other (*P*=0.71, Fisher test)^14^. In particular, note that independence would predict that 18.6% of MS cells would also be VS cells and that 8.8% of VS cells would also be MS cells. This is very similar to the proportions identified (17.0 and 8.0% respectively), leading to the conclusion that the expected proportion of cells that are both MS and VS cells is expected to be 1.6% (we found 20/1346, 1.5%). Such cells are of great interest, because they encode content-specific memories (i.e., ‘novel animal’ or ‘familiar landscape’). However, the calculation outlined above indicates that only a very large dataset such as the one released here allows investigation of this type of ‘memory engram’ cells.

## Usage Notes

### Description of code provided

Along with the data, we provide a comprehensive set of routines (all implemented in Matlab) that show how to read and process the data and how to perform basic single-unit analysis (located in the ‘Code/dataRelease/’ directory). The main routine is *NOneural_main_release.m*, and comments at the beginning indicate parameters that need to be adjusted before it can be run as-is. The list of sessions that form the dataset is provided in a structure NOsessions(NOID), which is defined in *defineNOsessions_release.m*. Each entry NOID (an index used throughout, see Table 1) defines the session-level parameters (which experimental variant was run, paths, session name, location of epileptic site). A list of NOIDs (called allSessionsToUse) is then used to determine which sessions are processed in a given analysis. The behavioral analysis is performed by *NO_behaviorSummary.m*, which summarizes behavior as shown in Fig. 1. The neural analysis is performed by *NOneural_loopOverSessions_release.m*, which in turn calls the single-neuron analysis function *NO_singleCellAnalysis_release.m* for every cell. This is achieved using *runForAllCellsInSession.m*, which calls a provided callback function for every cell in a session. *NO_singleCellAnalysis_release.m* holds the core single-neuron analysis and computes and plots all single-neuron metrics such as rasters and PSTHs (see Fig. 3). *EpilepticSitesExclusion.m* enables the user to exclude or include from analysis neurons according to whether they are recorded from within the epileptic focus or not. These pre-computed metrics are collapsed into the data structures totStats and cellStatsAll, which contain one entry per processed cell and are subsequently used for population-level analysis.

### Timestamps

All timestamps (spike and event times) are in units of μs, with an arbitrary (and unknown) starting point. All points of time are recorded by the acquisition system and thus no synchronization is needed between systems.

### Stimulus duration

The stimuli were shown on the screen for either 1 or 2 s, depending on the session. To determine for a given session how long the stimulus was shown, calculate the difference between the Stimulus On (1) and Stimulus Off (2) TTLs. Note that for the purposes of the analysis shown here this difference in stimulus presentation length does not have to be considered because it is relative to either stimulus or question screen onset. However, this difference has to be taken into account for stimulus offset aligned analysis.

## Additional information

**How to cite this article:** Faraut, M. C. M. *et al.* Dataset of human medial temporal lobe single neuron activity during declarative memory encoding and recognition. *Sci. Data* 5:180010 doi: 10.1038/sdata.2018.10 (2018).

**Publisher’s note:** Springer Nature remains neutral with regard to jurisdictional claims in published maps and institutional affiliations.

## Supplementary Material



## Figures and Tables

**Table 1:** Patients.

**ID**	**Number of sessions**	**Session ID(s)**	**Variant(s)**	**Age**	**Sex**	**Epilepsy Diagnosis**
H09	2	5, 6	1, 2	55	M	Right Mesial Temporal
H10	1	7	1	37	M	Left Frontal
H11	1	9	1	16	M	Right Lateral Frontal
H14	2	17, 18	2, 1	31	M	Bilateral Indep. Temporal
H15	2	20, 21	1, 2	45	M	Right Mesial Temporal
H16	2	23, 24	2, 1	34	F	Right Frontal
H17	1	32	1	19	M	Left Inferior Frontal
H18	1	26	1	40	M	Right Mesial Temporal
H19	2	27, 28	2, 1	34	M	Left Frontal
H21	3	38, 39, 41	2, 1, 3	20	M	Not Localized
H23	3	43, 44, 47	3, 2, 1	40	M	Left Mesial Temporal
H27	1	58	2	40	M	Bilateral Indep. Temporal
H28	1	48	3	22	M	Right Mesial Temporal
H29	1	49	3	17	F	Left Deep Insula
H31	1	50	2	30	M	Right Mesial Temporal
H33	1	52	1	29	M	Left Mesial Temporal
H42	1	54, 55	1, 1	29	M	Not Localized
H43	1	56	1	27	F	Left Mesial Temporal
H44	1	68	1	57	F	Right Mesial Temporal
H47	2	92, 97	1, 3	20	M	Right Mesial Temporal
H48	2	98, 99	2, 1	54	M	Left Mesial Temporal
H51	2	104, 105	3, 1	24	M	Bilateral Frontal and Temporal
C24	2	59, 60	1, 2	47	F	Not localized
C25	2	61, 63	1, 2	36	F	Bilateral Indep. Mesial Temporal
C26	2	64, 66	1, 2	56	F	Left Mesial Temporal
C27	1	67	1	44	M	Left Mesial Temporal
C29	2	69, 70	1, 2	19	M	Left Neocortical Temporal
C31	1	73	3	32	M	Left Neocortical Temporal
C32	1	74	2	19	M	Not Localized (Generalized)
C33	3	76, 77, 78	3, 2, 1	44	F	Right Mesial Temporal
C34	1	85	3	70	M	Bilateral Mesial Temporal
C37	1	96	3	33	F	Right Mesial Temporal
C38	1	102	3	63	F	Right Mesial Temporal
C39	2	93, 100	1, 3	26	M	Right Insula
C40	1	101	3	25	M	Right Motor Cortex
C42	2	111, 112	1, 2	25	F	Not Localized
C43	1	113	3	42	F	Left Mesial Temporal
C44	1	114	3	53	F	Right Mesial Temporal
C47	1	115	3	32	M	Right Mesial Temporal
C48	1	116	3	32	F	Left Mesial Temporal
C49	2	117, 118	3, 1	24	F	Left Mesial Temporal
C51	2	119, 120	3, 1	17	M	Not Localized (No Seizures)
Total Subjects: 42		Total Sessions: 65		Total Female: 15		Total Male: 27
List of number of sessions performed by patients, the designated ID and variants for each session, patient demographics, and pathology. For epilepsy diagnosis, mesial temporal refers to the complex of mesial structures including amygdala and hippocampus, and parahippocampal gyrus. Neocortical temporal refers to the lateral gray matter.						

**Table 2:** The files and visual categories used in each of the three variants of the experiment.

**Variant**	**Stimulus file**	**Experiment file**	**Images directory**	**Categories (Category ID)**
var1	newOldDelayStimuli.mat	NewOldDelay_v3.mat	newolddelay	Houses (1), Landscapes (2), Mobility/Vehicles (3), Phones/Objects (4), Animals (5)
var2	newOldDelayStimuli2.mat	NewOldDelay2_v3.mat	Newolddelay2	Fruit (1), Kids/People (2), Military/Vehicles (3), Space (4), Animals (5)
var3	newOldDelayStimuli3.mat	NewOldDelay3_v3.mat	Newolddelay3	Cars (1), Food (2), People (3), Spatial (4), Animals (5)

**Table 3:** Number of cells recorded and spike sorting quality metrics.

**Area**	**# cells recorded**	**%ISI<3 ms ±s.d.**	**Mean rate ±s.d.**	**SNR ±s.d.**	**CV2 ±s.d.**	**Burst index ±s.d.**	**Spike width**
							**[d±s.d., P uni]**
Hippocampus	633	0.33±0.5	2.23±3.2	2.01±1.4	0.95±0.2	0.04±0.06	0.54±0.26, *P*=0.0004
Amygdala	943	0.33±0.5	2.04±2.7	2.13±1.2	0.97±0.2	0.04±0.06	0.62±0.29, *P*=0.0004
All errors are ±s.d. P indicates the *P*-value for the Hartigan DIP test testing the unimodality of the spike width distribution (with 2500 bootstrap runs).							

**Table 4:** Number of cells in each area selected as MS, VS and their electrophysiological characteristics.

**Area**	**# (%) of VS cells**	**# (%) of MS cells**	**Firing rate VS [Hz ±s.d.]**	**Firing rate MS [Hz ±s.d.]**	**Spike width VS [d ±s.d., P uni]**	**Spike width MS [d ±s.d., P uni]**
Hippocampus	94 (17)	58 (11)	2.4**±**0.3	2.8**±**0.4	0.58±0.03, *P*=0.0004	0.53±0.03, *P*=0.002
Amygdala	156 (20)	60 (8)	2.2**±**0.2	2.1**±**0.3	0.62±0.02, *P*=0.04	0.58±0.04, *P*=0.47
All errors are ±s.d. P indicates the *P*-value for the Hartigan DIP test testing the unimodality of the spike width distribution (with 2,500 runs).						
